# Pollen Morphology and Boron Concentration in Floral Tissues as Factors Triggering Natural and GA-Induced Parthenocarpic Fruit Development in Grapevine

**DOI:** 10.1371/journal.pone.0139503

**Published:** 2015-10-06

**Authors:** Orlando Alva, Rosa Nair Roa-Roco, Ricardo Pérez-Díaz, Mónica Yáñez, Jaime Tapia, Yerko Moreno, Simón Ruiz-Lara, Enrique González

**Affiliations:** 1 Instituto de Ciencias Biológicas, Universidad de Talca, Talca, Chile; 2 Instituto de Química de Recursos naturales, Universidad de Talca, Talca, Chile; 3 Centro Tecnológico de la Vid y el Vino, Facultad de Ciencias Agrarias, Universidad de Talca, Talca, Chile; University of Minho, PORTUGAL

## Abstract

Parthenocarpic fruit development (PFD) reduces fruit yield and quality in grapevine. Parthenocarpic seedless berries arise from fruit set without effective fertilization due to defective pollen germination. PFD has been associated to micronutrient deficiency but the relation of this phenomenon with pollen polymorphism has not been reported before. In this work, six grapevine cultivars with different tendency for PFD and grown under micronutrient-sufficient conditions were analyzed to determine pollen structure and germination capability as well as PFD rates. Wide variation in non-germinative abnormal pollen was detected either among cultivars as well as for the same cultivar in different growing seasons. A straight correlation with PFD rates was found (R^2^ = 0.9896), suggesting that natural parthenocarpy is related to defective pollen development. Such relation was not observed when PFD was analyzed in grapevine plants exposed to exogenous gibberellin (GA) or abscissic acid (ABA) applications at pre-anthesis. Increase (GA treatment) or reduction (ABA treatment) in PFD rates without significative changes in abnormal pollen was determined. Although these plants were maintained at sufficient boron (B) condition, a down-regulation of the floral genes *VvBOR3* and *VvBOR4* together with a reduction of floral B content in GA-treated plants was established. These results suggest that impairment in B mobility to reproductive tissues and restriction of pollen tube growth could be involved in the GA-induced parthenocarpy.

## Introduction

Grapevine is one of the most cultivated and economically important fruit crop worldwide. Even when grapes are used for multiple purposes, wine produced from the different *Vitis vinifera* cultivars has the highest economic value. Fruit yield and quality are essential for winemaking and some cultivars including *Vitis vinifera* cv. Carménère, widely cultivated in Chilean vineyards, exhibit high tendency to fruitlet abscission and “millerandage”, reproductive disorders that seriously affect these traits. In grapevine as well as in other plant species, fruitlet abscission is correlated with an unusual polyamine content in fruits at setting stage, being spermidine a key regulator of physiological abscission by modulating the sugar and amino acid contents in developing inflorescences [1–4]. On the other hand, “millerandage” is characterized by the presence of normal size seeded berries together with small-size (<3mm) and mid-size (3–6mm) seedless fruits in the same bunch. While small seedless berries (commonly referred as “shot berries”) are presumably generated from defective, non-fertile ovules, mid-size seedless berries arise from a parthenocarpic event caused by defective ovule fertilization due to failure in pollen tube growth and sperm cells release into ovaries [5,6]. Although little is known about the factors triggering PFD, some authors associate this phenomenon with deficiency in essential micronutrients such as boron (B) and zinc (Zn) [7–10]. In plants, B is involved in the cross-linking of two rhamnogalacturonan II (RG-II) chains, a cell wall pectic polysaccharide required for pollen tube development [11]. Therefore, B deficiency can inhibit reproductive growth by affecting pollen germination, pollen tube growth, fruit set and seed formation [12–14]. On the other hand, Zn is required as a cofactor for over 300 enzymes and proteins involved in cell division, nucleic acid metabolism and protein synthesis, and is critical in the control of gene transcription and the coordination of other biological processes regulated by proteins containing DNA-binding Zinc-finger motifs, RING fingers and LIM domains [15,16]. Foliar applications of B and Zn to maintain adequate micronutrient concentrations is a common vineyard management practice [17,18].Nonetheless, variable PFD rates are still detected after these treatments, suggesting that causes other than micronutrient deficiency are also originating this phenomenon. In this sense, since changes in endogenous levels of GA, ABA and auxins after pollination are determining factors for fruit setting and initiation [19,20], hormonal causes for PFD have been also invoked. Fruit set occurs even without pollination by inducing the expression of genes associated to GA and auxin biosynthesis [20]. Supporting this assumption, exogenous GA application to inflorescences at pre-bloom stage can induce fruit set without effective fertilization, leading to a PFD in different grapevine cultivars [19,21–23]. Changes in expression of genes involved in both GA and auxin signaling pathways leading to the down-regulation of genes coding for putative fruit initiation repressors were determined [24].

Pollen quality and its germination potential are essential for fertility. Pollen polymorphism is a widespread phenomenon among higher plants including different grapevine species [25–29]. Particularly, in some low productivity grapevine cultivars, the normal tricolporate pollen is mixed with structurally aberrant grains showing bicolporate, acolporate, collapsed or shriveled morphology [26,30–32]. However, the relation between abnormal pollen occurrence and fruitlet abscission or PFD has been not investigated. Taking this into account, in this study we analyze the morphology and germination capability of pollen together with the abscission and PFD rates. In a first approach, such parameters were studied in six field-grown grapevine cultivars selected by their different tendency to present abscission and PFD but maintaining a sufficient B and Zn nutritional status. In a second approach, we focused on *Vitis vinifera* cv. Carménère because its relevance for Chilean wine industry. Abnormal pollen, PFD rates and floral B content were determined in GA and ABA-treated plants also cultivated under sufficient B and Zn. Results suggest that while impairment in pollen development and the incidence of abnormal pollen could be the main cause of parthenocarpy under field-growing conditions, variation in PFD rates observed in GA and ABA exposed plants seems to be related to changes in transporter-mediated B mobility to floral tissues.

## Material and Methods

### Plant material

Mature pollen grains from six certified ampelographic collection of *Vitis vinifera* cultivars (Cabernet Sauvignon, Carménère, Chardonnay, Merlot, Malbec and Syrah) were obtained during two consecutive seasons (S1 and S2) from plants growing under field conditions in a vineyard located at the Estación Experimental Panguilemo, Universidad de Talca (Maule Valley, Central Chile, 35°22.2’ S, 71°35.39’ W, 121 m.a.s.l.). The region is characterized by mediterranean climatic conditions with approximately 1800 growing degree days (Winkler III) with a dry season of 6 months and an average rainfall of 550 mm concentrated during the winter period. The vineyard soil has a clay loam texture and a slope of about 1%. The vines were planted in 1998 with a spacing of 1.5 m x 3,0 m (2,222 vines/ha) on their own roots and were trained to a vertical shoot positioned system (VSP) with East-West oriented rows and flood-irrigated. Nutritional status of plant leaves was monitored and corrected by foliar spray applications to maintain Zn (45–55 ppm) and B (55–75 ppm) sufficiency conditions. Developmental stages to be sampled were defined according to the Eichorn-Lorenz scale [33]. Fresh pollen grains were collected at full bloom (EL23) and used directly or stored at –80°C for later use. For PFD and abscission measurements, bunches were collected at véraison stage (EL35). For gene expression analysis, developmental stages collected were: EL19, flowers at pre-anthesis; EL23, flowers at anthesis; EL29, fruits at setting; EL31, berries at pre-véraison; EL35, berries at véraison.

### Determination of abscission and PFD rates

Three randomly chosen clusters from each of six randomly chosen plants per each cultivar were marked and the number of total flowers at full bloom (EL23) was counted. At véraison stage (EL35), total grapes and mid-size seedless berries (3–6 mm in diameter) in each cluster were determined. Abscission rate was estimated as the ratio between total grapes against total flowers, while PFD rate was calculated as the ratio between mid-size seedless berries against total grapes in a bunch.

### Pollen structure analysis

Exine structure of pollen grains was examined in acetolyzed samples processed according to the method described by Erdtman [34] using an Olympus BX51 microscope coupled to Olympus C–7070 digital camera. Several fields per sample were photographed and around a thousand of individual pollen grains were classified in order to calculate the exine abnormality ratio. Pollen was also analyzed by scanning electron microscopy (SEM). The samples were dehydrated in ethanol and dried at the critical point. After that, pollen grains were coated with a gold film using a JEOL FC 1100 coater and examined with a JEOL/JEM 1200 EX II scanning electron microscope.

### Pollen germination assays

Pollen grains were distributed in 6 cm diameter Petri dishes containing basal germination medium composed by 1mM CaCl_2_, 15% sucrose, pH 5.8 and 1% agar and incubated under darkness at 25°C for 24 h [35]. In order to evaluate the effect of B on pollen germination, basal germination medium was supplemented with 1 mM boric acid. To calculate the germination rate, three pollen containing dishes per each cultivar were examined under light microscopy and over 600 pollen grains per cultivar sample were analyzed for germination capacity and exine structure. Pollen grains were estimated as germinated when the length of the pollen tube exceeded the pollen grain diameter.

### Hormonal treatments and analysis

Five-year-old clonally propagated plants of *Vitis vinifera* cv. Carménère growing under the field condition described in Plant material were used for exogenous treatment with GA and ABA. Experiment was carried out as described before [24] with minor modifications. Seven days before anthesis, inflorescence clusters were immersed for 30 s in a solution containing either 290 μM GA_3_ (PhytoTechnology Laboratories, USA) or 100 μM ABA, (PhytoTechnology Laboratories, USA) both dissolved in 100 UI/L of Silwet L77 (Sigma-Aldrich, USA) as surfactant. For control plants, inflorescences were treated with a solution containing only the surfactant. Three plants were employed for each treatment. Mature pollen grains were collected at anthesis from two randomly chosen clusters from each plant and processed to analyze pollen structure as described previously. B concentration in flowers collected at anthesis was determined as described by Pérez-Castro et al [36]. PFD rates from treated and control plants were determined as previously indicated in four randomly chosen clusters collected at véraison stage.

### Gene expression analysis

For expression analyses, four randomly chosen clusters from different plants were independently processed for RNA isolation (biological replicates). Total RNA was extracted from 2 to 3 g of frozen material at the stages defined in Plant Material, using the modified CTAB method [37]. Three independent extractions were made from each sample and RNA integrity analysis and quantification were carried out by using Agilent RNA 6000 Nano Kit for the Agilent 2100 Bioanalyzer System. Following DNase (DNAse I, Ambion) treatment of total RNA, first-strand cDNA synthesis was carried out from 2 μg of total RNA for each sample using oligo (dT) according to the manufacturer’s instructions (AffinityScript QPCR cDNA Synthesis Kit, Stratagene, La Jolla, CA). Quantification of transcripts by real-time quantitative reverse transcription–PCR (qRT–PCR) was performed as described previously by Almada et al [38]. Expression was normalized to the *V*. *vinifera* glyceraldehyde phosphate dehydrogenase (*GAPDH*) gene (*VvGAPDH*; GenBank database accession CN938023) and *Ubiquitin* gene (*VvUBQ*, TIGR database accession TC32075). Specific primers were designed for each gene with the software PrimerQuest from Integrated DNA Technologies, Inc. (https://www.idtdna.com/Primerquest/Home/Index). Oligonucleotide sequences were:

*VvBOR1*Fwd, 5’-ACGCTTGAATGAGTTGAGG–3’

*VvBOR1*Rev, 5’-GCTTGAAGACGAACCATGAGG–3´

*VvBOR3*Fwd, 5’-GAGAAGCAAGACAGTCCAATTTAGG–3´

*VvBOR3*Rev, 5’-AAAGCCCACATCAAGGAAGAGG–3´

*VvBOR4*Fwd, 5’-ACCAGTCGGACGCAGTGAG–3´

*VvBOR4*Rev, 5’-AGGTGTAATCAGTGGACTCTATACG–3´

*VvGAPDH*Fwd, 5’-TTCCGTGTTCCTACTGTTG–3’

*VvGAPDH*Rev, 5’-CTCTGACTCCTCCTTGAT–3’

*VvUBQ* Fwd, 5´- GTG GTA TTA TTG AGC CAT CCT T–3´

*VvUBQ* Rev, 5´- AAC CTC CAA TCC AGT CAT CTA–3´

### Statistical analysis

Analysis was carried out with IBM SPSS Statistics V20.0 for Windows; the statistical comparison included analysis of variance, standard error and correlation analysis. Data were compared by Student´s t-test. Least significance difference was calculated at *p<0*.*05*.

## Results

### Abnormalities in pollen morphology were detected in different field growing grapevine cultivars

**Fig 1:**
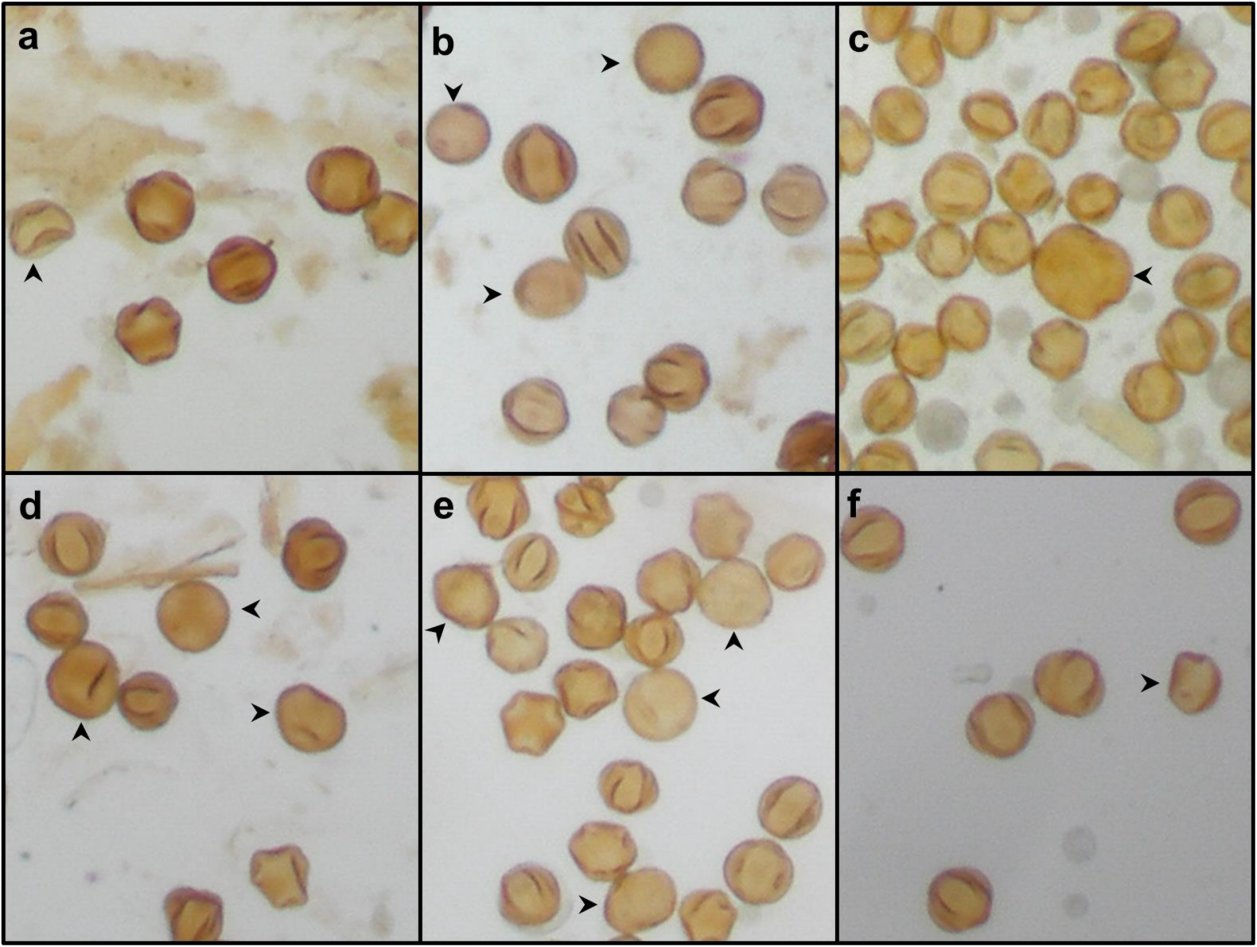
Acetolyzed pollen grains from six *Vitis vinifera* cultivars analyzed under light microscopy. a) Cabernet Sauvignon, b) Carménère, c) Chardonnay, d) Malbec, e) Merlot and f) Syrah. Samples were visualized at 40X magnification. Abnormal pollen grains are pointed by arrowheads.

Pollen structure was determined for six *V*. *vinifera* cultivars grown on the same field and exposed to identical environmental conditions and agronomical management. Cultivars were examined in either one growing season (for Chardonnay, Malbec and Syrah) or two growing seasons (for Cabernet Sauvignon, Carménère and Merlot). To examine the pollen morphology, grains were collected from the above mentioned cultivars and processed for analysis. Light microscopy inspection of acetolyzed pollen samples shows that in all varieties, most of pollen corresponds to the normal tricolporate type with evident furrows and without significant differences in size. Additionally, variable amounts of malformed pollen grains exhibiting a diversity of shapes and sizes were also found in all analyzed cultivars (Fig 1).

**Fig 2:**
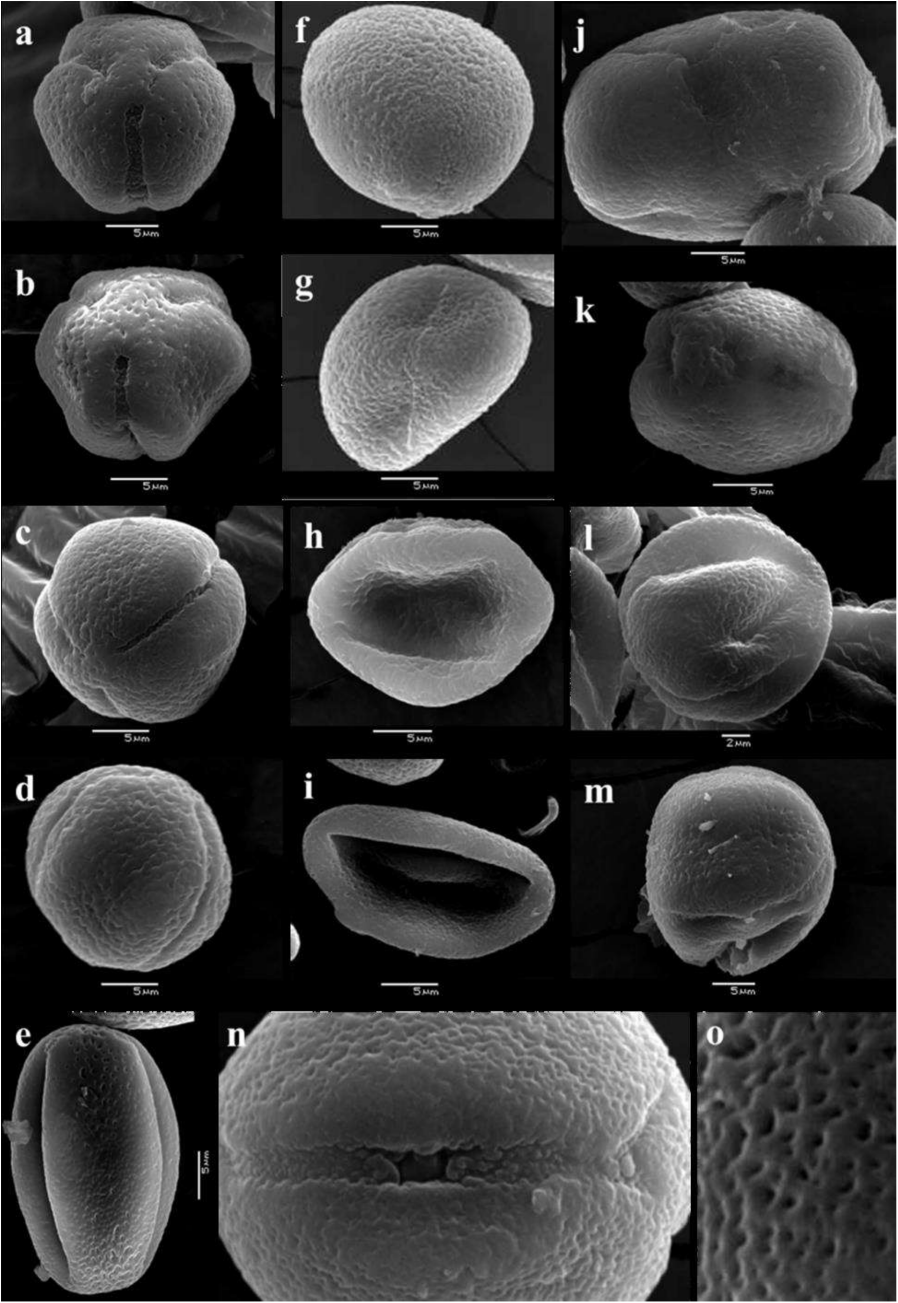
Morphology of normal (a-e,n,o) and abnormal (f-m) pollen grains from six *Vitis vinifera* cultivars analyzed under scanning electron microscopy. a) Cabernet sauvignon (polar view); b) Chardonnay (sub-polar view); c) Malbec (sub-equatorial view); d) Carménère (equatorial view); e) Syrah (equatorial view); f) Carménère acolporate; g) Merlot acolporate; h) Carménère acolporate and collapsed; i) Malbec acolporate and collapsed, j) Carménère jumbo; k) Malbec bicolporate; l) Merlot irregular; m) Carménère irregular; n) Carménère anatomy of endo (germinal pore) and ectoapertures (colpi); and o) Cabernet sauvignon classical exine sculpture.

For a more detailed analysis, pollen samples were also visualized under scanning electron microscope. With the exception of Syrah, normal pollen from all cultivars appeared as subspherical, trilobated and radially symmetrical without significant morphological differences among the cultivars (Fig 2A–2D). Syrah normal pollen grains are also symmetrically trilobated but show an elongated form in the equatorial view (Fig 2E). Ectoapertures of pollen grains of all analyzed cultivars are narrow and long whereas endoapertures are circular (Fig 2N). Different types of abnormal pollen: acolporate, bicolporate, collapsed, irregular, small and bigger grains (Fig 2F–2M) were found in all cultivars, being the acolporate type (spheroidal shape without furrows or germinative pores) the most common abnormal pollen in those cultivars with higher tendency for PFD (Carménère, Merlot and Malbec). Abnormal pollen shows similar exine sculpture as that in normal grains (Fig 2O).

### Abnormal pollen is defective in germination and pollen tube growth

**Fig 3:**
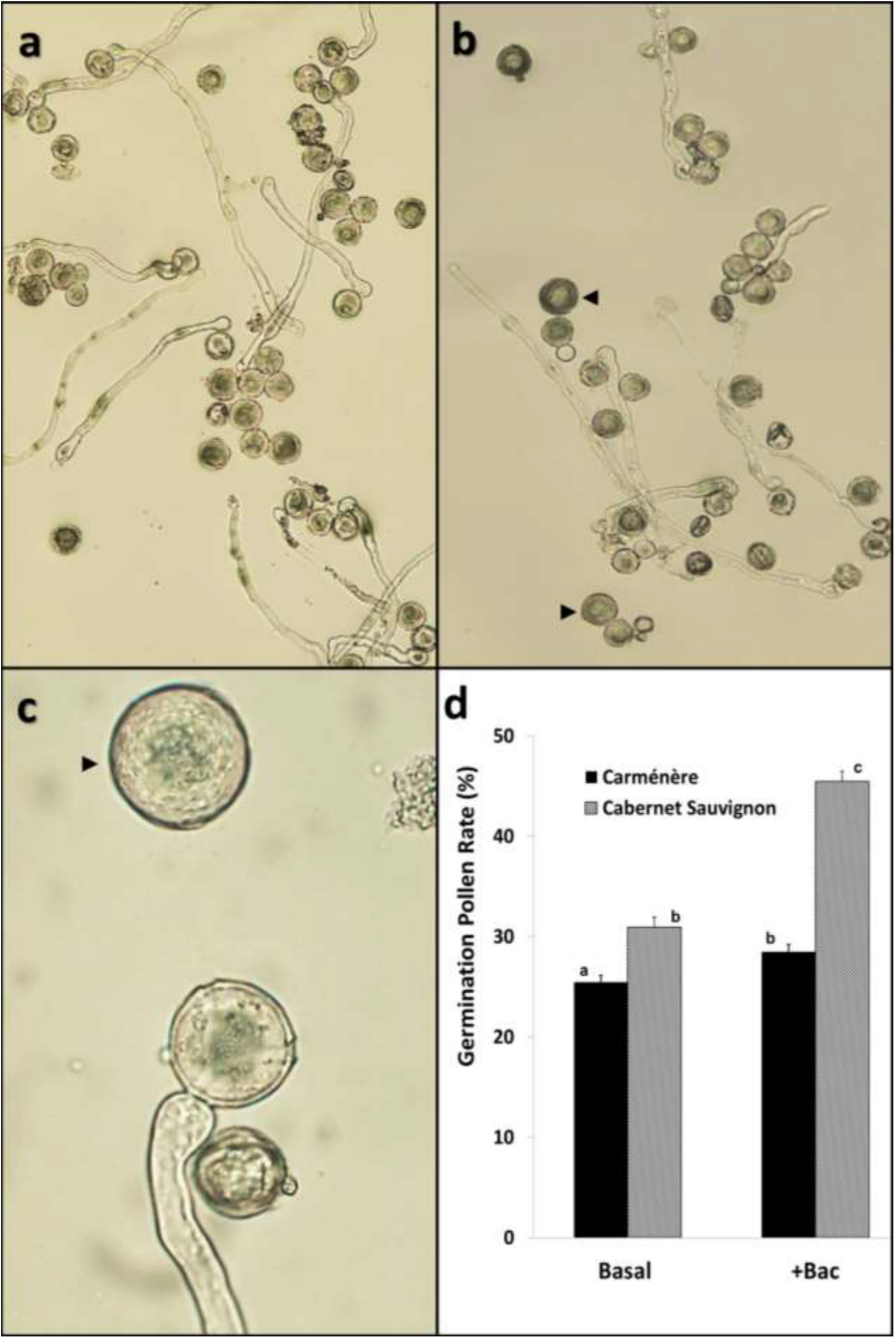
Germination capability of pollen grains from two *V*. *vinifera* cultivars examined under light microscopy. a) Cabernet Sauvignon in basal media (100x); b) Carménère in basal media (100x), c) Carménère in basal media (400x); and d) Germination rates in either basal or 1mM borate supplemented media at 25°C. Acolporate pollen grains in b) and c) are pointed by arrows. Basal: basal germination medium composed by 1mM CaCl2, 15% sucrose, pH 5.8 and 1% agar. +Bac: basal germination medium supplemented with 1mM borate. Means with different letters in d) are significantly different at p<0.05.

To evaluate pollen germination capability two cultivars with different susceptibility to PFD were selected: Cabernet Sauvignon (low tendency to PFD) and Carménère (high tendency to PFD). Pollen was incubated in basal germination media and near 600 normal and abnormal grains from each cultivar were examined. Pollen tube growth was detected only in normal pollen from Cabernet Sauvignon (Fig 3A) and Carménère (Fig 3B and 3C). Neither acolporate grains nor other abnormal types of pollen were able to germinate (black arrows in Fig 3B and 3C). Under the assayed conditions, germination rate of Cabernet Sauvignon pollen was higher than that determined for Carménère (34.9% vs. 28.2%; Fig 3D). Since it has been reported that B addition improves pollen germination in several plant species [39,40], pollen grains were also incubated in germination media supplemented with 1mM sodium borate. A significant increase in germination rate of normal grains from Cabernet Sauvignon (48.5%) was detected but only a minor effect on Carménère pollen (29.3%) was observed, suggesting functional differences between normal pollen from the two cultivars.

### PFD rates correlate with abnormal pollen rates in field grown grapevine cultivars

**Fig 4:**
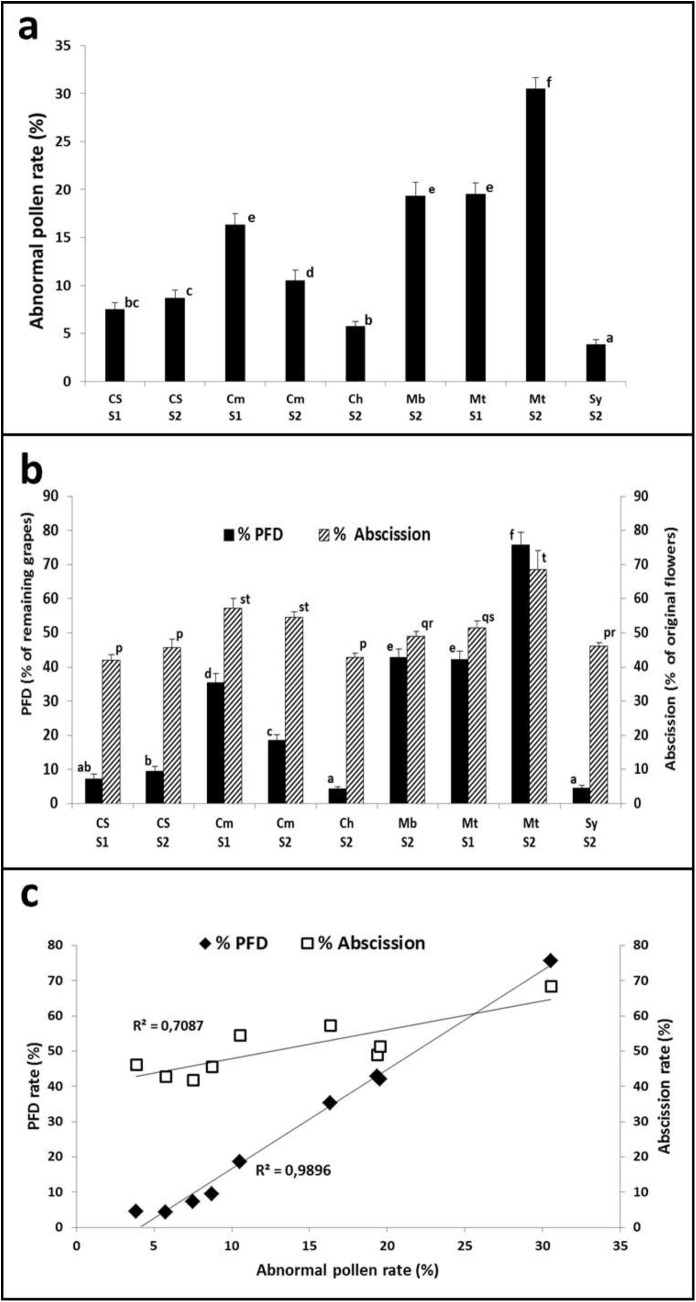
Quantification of abnormal pollen, millerandage and fruitlet abscission rates in the *V*. *vinifera* cultivars Cabernet Sauvignon (CS), Carménère (Cm), Chardonnay (Ch), Malbec (Mb), Merlot (Mt) and Syrah (Sy) in two growing seasons (S1 and S2). a) Abnormal pollen rates determined in samples processed by the Erdtman acetolysis method; b) PFD and fruitlet abscission rates; and c) Correlation between abnormal pollen and PFD rates (black squares) and between abnormal pollen and fruitlet abscission rates (open squares). Means with different letters are significantly different at p<0.05.

When the rate of abnormal pollen was estimated, significant differences were detected among varieties as well as for the same cultivar between growing seasons S1 and S2 (Fig 4A). Higher rates were determined for cultivars with high tendency to present abscission and parthenocarpy as Merlot (30% in S2 and 18% in S1), Malbec (18%) and Carménère (16% in S1 and 10% in S2), whereas varieties characterized by their low tendency to these reproductive disorders such as Cabernet Sauvignon, Chardonnay and Syrah, showed lower rates of aberrant pollen (<10%). On the other hand, abscission rates varied from 41% (Cabernet Sauvignon S1) to 68% (Merlot S2). Among those cultivars examined during two growing seasons, significant differences in abscission rates were found only for Merlot (Fig 4B). No direct relation between abscission and abnormal pollen rate was observed (Fig 4C). PFD rates ranged from 5% to 75% in the examined cultivars. Those cultivars with low tendency to parthenocarpy (Chardonnay, Syrah and Cabernet Sauvignon) exhibited PFD rates lower than 10% whereas cultivars with high tendency to parthenocarpy (Carménère, Malbec and Merlot) displayed PFD rates over 18%. When analyzed, remarkable variations between the two growing seasons were found for some cultivars: i.e. PFD rates in Carménère dropped from 35% in S1 to 18% in S2, while the same trait in Merlot scaled from 42% in S1 to 75% in S2 (Fig 4B). A straight correlation (R^2^ = 0.9896) between PFD and abnormal pollen rates was established when data collected from S1 and S2 growing season were used (Fig 4C). Such correlation was the same when the analysis was performed considering only the data for the 6 cultivars in S2 growing season or considering only the data from the 3 cultivars examined in S1 and S2 growing seasons (R^2^ = 0.9901 and R^2^ = 0.9967, respectively; data not shown). These results suggest that the presence of abnormal pollen grains could be the main cause of the parthenocarpic phenomenon when grapevine plants are grown under B and Zn sufficient conditions.

### In GA-induced parthenocarpy, B concentration in floral tissues but not abnormal pollen rates is affected

Induction of parthenocarpy in grapevine by exogenous GA application provides an adequate model for the study of this phenomenon. To test whether GA-induced PFD is also due to an increase in abnormal pollen rates, inflorescence clusters (7 days before anthesis) of clonally propagated *V*. *vinifera* cv. Carménère plants, were treated with GA at 290 μM. Abnormal pollen and PFD rates were determined (Table 1). While in non-treated control plants the PFD rate was estimated as 29.6 ± 2.8%, in the GA-treated plants a marked increase in PFD was observed (69.3 ± 7.2%). On the other hand, when inflorescence clusters were exposed to 100 μM ABA, a decrease in PFD rate was observedd (22.7 ± 3.7%). However, the analysis of pollen morphology did not reveal significant differences in abnormal pollen rates between control (9.1 ± 0.9%) and hormone-treated plants (11.4 ± 1.2% and 10.3 ± 0.6 for GA-treated and ABA treated plants, respectively) and therefore no correlation was established between PFD and abnormal pollen rates.

**Table 1:** Effect of hormonal treatments on PFD rates, abnormal pollen rates and boron content in floral tissues from *Vitis vinifera* cv Carménère.

Treatment	PFD rate (%)	Abnormal Pollen rate (%)	Boron content (μg/g dry weight)
**Control**	**29.6 ± 2.8**	**9.1 ± 0.9**	**14.3 ± 0.3**
**ABA**	**22.7 ± 3.7**	**10.3 ± 0.6**	**22.9 ± 3.4**
**GA**	**69.3 ± 7.2**	**11.4 ± 1.2**	**5.5 ± 0.2**

Taking into account the role of borate in pollen germination and pollen tube growth, the B content in grapevine flower at anthesis was determined in both control and hormone- treated plants (Table 1). When compared to control non-treated plants, ABA and GA have opposite effects, while B concentration increased 1.6-fold in ABA-treated plants, it was reduced 2.6-fold when exposed to exogenous GA.

### The expression of grapevine BOR-type transporter encoding genes are modified in response to exogenous ABA and GA applications

Since BOR-type B transporters are essential for B mobility in plants [41,42], the involvement of such transporters in GA-induced parthenocarpy was investigated. Previously, six genes encoding putative BOR-type transporters grouped in two phylogenetic clades have been identified in the grapevine genome and one of them, *VvBOR1*, was fully characterized [36]. Protein sequences encoded by *VvBOR* genes were obtained from the GENOSCOPE database (http://www.genoscope.cns.fr/externe/GenomeBrowser/Vitis/) and compared to AtBOR1 protein as reference (Table 2). Based on the percentage of identity to AtBOR1, the remaining genes will be referred as *VvBOR2* (NCBI RefSeq XP_002272575.1) and *VvBOR3* (NCBI RefSeq XP_002263974), which encodes proteins grouped in the same clade with AtBOR1; and *VvBOR4* (NCBI RefSeq XP_002282436.1), *VvBOR5* (NCBI RefSeq XP_002285279.1) and *VvBOR6* (NCBI RefSeq XP_002281778.2) which encodes transporters clustered together with AtBOR4 [36].

**Table 2:** Grapevine *VvBOR* gene family and identity of encoded proteins with the reference protein AtBOR1 from *Arabidopsis thaliana*.

Gene	Chromosome	NCBI RefSeq	Protein size	% Identity to AtBOR1
*VvBOR1*	17	XP_002282501	720 aa	80.25%
*VvBOR2*	7	XP_002272575.1	718 aa	74.96%
*VvBOR3*	5	XP_002263974	721 aa	74.79%
*VvBOR4*	4	XP_002282436.1	669 aa	55.0%
*VvBOR5*	9	XP_002285279.1	668 aa	54.59%
*VvBOR6*	11	XP_002281778.2	675 aa	54.16%

**Fig 5:**
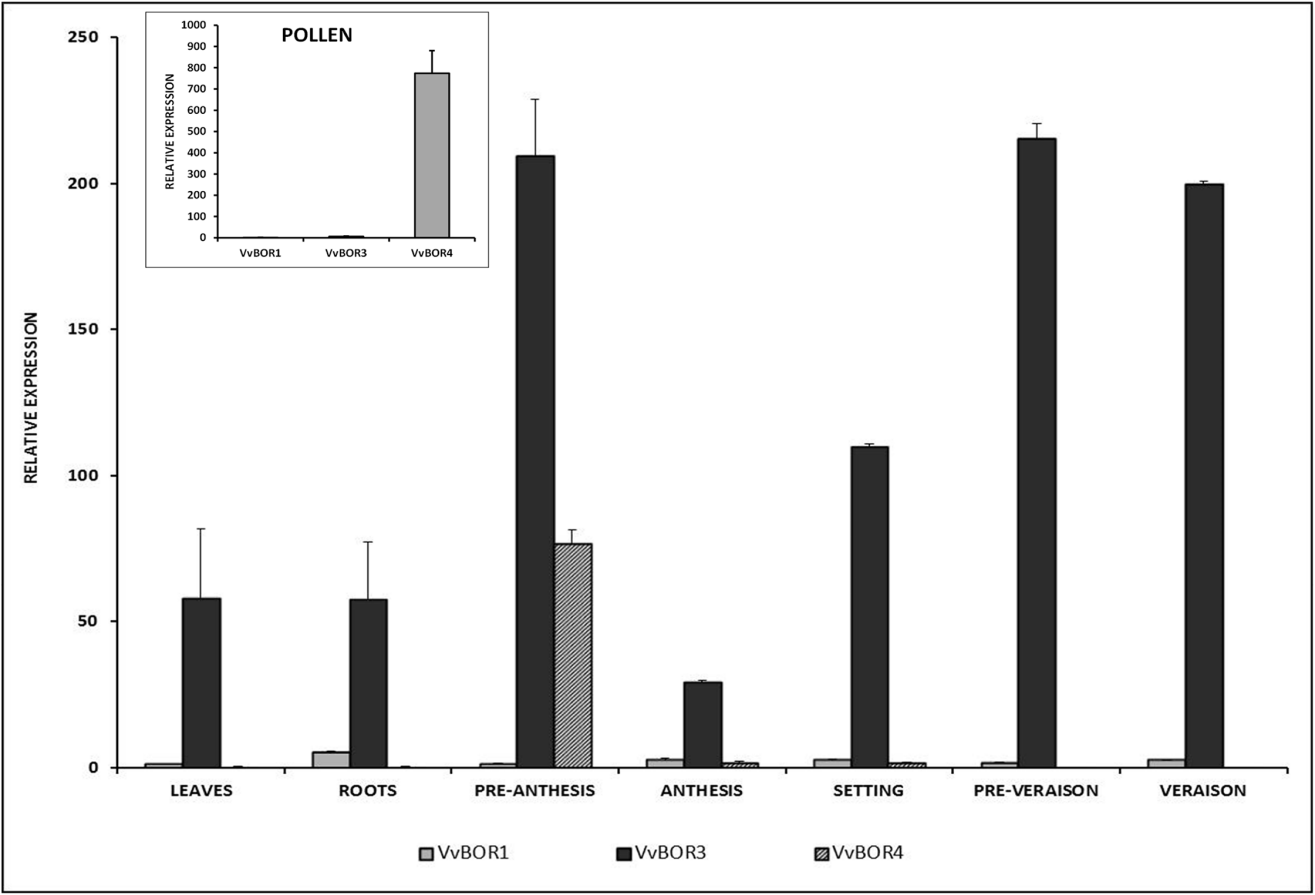
Expression profiles of *VvBOR1*, *VvBOR3* and *VvBOR4* genes in vegetative (leaves and roots) and reproductive organs of *Vitis vinifera cv*. *Carménère*. Pre-anthesis (flowers, 5 days before full bloom), anthesis (pollen depleted flowers at full bloom), setting (fruits, 2 days after pollination), pre-véraison (berries, 2 weeks after pollination) and véraison (berries, 8 weeks after pollination). The insert represent expression levels in pollen from flowers at anthesis. *VvBOR1* expression in leaves was adjusted to 1 relative unit. Data represent the means of 4 biological replicates ± SD.

Expression profiles of *VvBOR* genes in different organs and reproductive developmental stages were analyzed and compared with *VvBOR1* transcription pattern. Two of these genes, *VvBOR3* and *VvBOR4*, were found to be strongly expressed in flowers at pre-anthesis stage, however differences in tissue-specific expression at full bloom (anthesis) and later developmental stages were observed. While *VvBOR*3 is transcribed in pollen-depleted flowers at anthesis and in developing berries, *VvBOR4* displays a pollen-specific expression mode (Fig 5). Compared to *VvBOR1*, the expression of *VvBOR2*, *VvBOR5* and *VvBOR6* was not substantial in reproductive tissues (data not shown).

**Fig 6:**
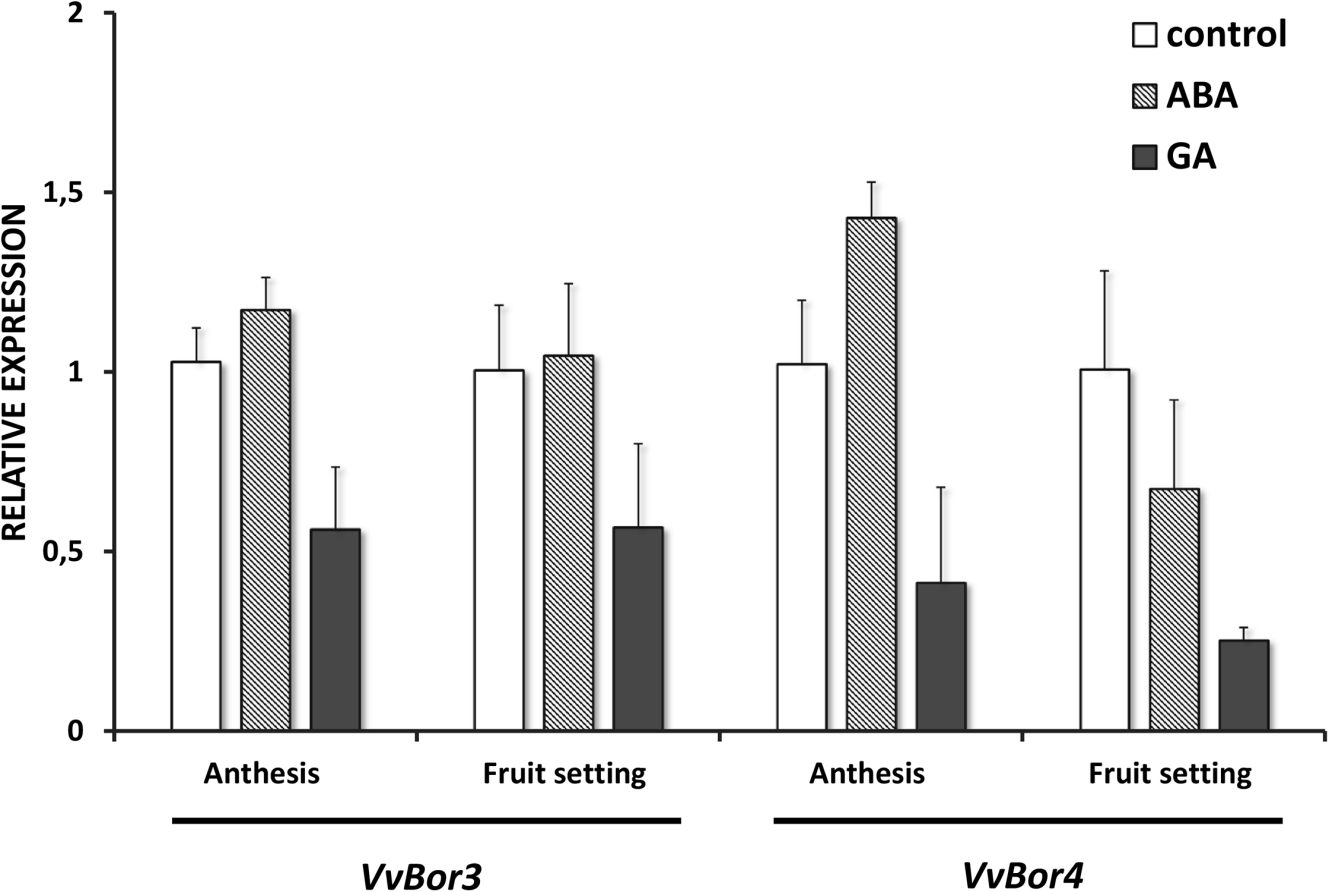
*VvBOR3* and *VvBOR4* expression in response to hormone applications. Analysis was performed on flowers at anthesis and fruits at setting from *Vitis vinifera* cv Carménère plants exposed to 100 μM ABA or 290 μM GA. Non-treated plants were used as a control. For each determination, expression of the respective gene in control samples was adjusted to 1 relative unit. Data represent means of 4 biological replicates ± SD.

The transcriptional activity of both genes was determined in flowers at anthesis and at fruit setting stages from control and hormone-treated Carménère plants. A significative down-regulation of *VvBOR3* and *VvBOR4* expression in response to exogenous GA was detected in both stages (Fig 6). On the other hand, ABA treatments did not significantly affect *VvBOR3* expression, but a moderate induction of *VvBOR4* expression was observed at anthesis stage (Fig 6). The above results indicate that the observed changes in floral B concentration determined for GA- and ABA-treated plants could be caused by the modulation of the referred BOR-type B transporter encoding genes.

## Discussion

### Pollen polymorphism is a widely distributed trait among different grapevine cultivars

Structurally aberrant pollen has been detected in different field grown grapevine cultivars from a commercial vineyard. The most abundant abnormal pollen corresponds to the acolporate type (without furrows and germination pores) but other forms (collapsed, bicolporate, irregular in size) were also observed. These pollen grains were not able to germinate when assayed in basal media, suggesting that they are not functional. The occurrence of morphologically abnormal pollen (mostly of the acolporate type) has been also observed in other grapevines cultivars like Loureiro, Mourisco, Picolit Giallo, Moscato Rosa, Ceresa and Bicane, all of which are characterized by their low fruit productivity [30–32]. Interestingly, this kind of grains resembles the pollen found in female flowers of dioecious vines like *V*. *coignetiae*, *V*. *riparia*, *V*. *aestivalis* or the wild *V*. *vinifera* ssp. *silvestris*, which display round shape, lack of germination structures (colpi) and are frequently collapsed. In these species, pollen from female plants is non-functional and is unable to germinate on basal germination media [29,43,44]. The appearance of hermaphrodite flowers, one of the most important traits developed during grapevine domestication, has been suggested to be the result of a mutation, and the development of acolporate pollen could be some reminiscence of their earlier dioecious lineages [45,46]. Supporting this idea, studies on the dioecious *V*. *amurensis* var. Rupr demonstrate that application of N1-(2-chloro-4-pyridyl)-N3-phenylurea (CPPU), can convert male flowers to a hermaphrodite-like type and proteomic analysis revealed 17 proteins participating in the sex conversion phenomenon [47]. In these plants, besides stimulation of pistil development, pollen germination rate was lower than in control and such difference could be attributed to the presence of some rounded pollen grains without any observable colpi in treated plants [48].

### Pollen morphology is the main determining factor for PFD in grapevine plants growing under conditions of sufficient micronutrients

Fruitlet abscission and PFD, among others, are determining factors for low productivity in some grapevine cultivars [5,6]. However, the relationship between these reproductive disorders and the presence of structurally defective pollen has not been established before. Abnormal pollen rates and PFD rates determined for the examined cultivars showed a wide variation among cultivars and for the same cultivar in different growing seasons, and a straight correlation has been observed between the two traits. Since other parthenocarpy-promoting factors (i.e. micronutrient deficiency) are absent in these plants, pollen quality and its germination capability could be the main cause of PFD when grown in the field. Hence, great attention should be given to pollen development and its regulation when studying PFD. In this sense, studies on pollen morphogenesis in the model plant *Petunia hybrida*, show that *tapetum* and pollen development are well coordinated processes modulated by several anther-specific “zinc finger” (ZnF) transcription factors of the ZPT-family [49]. Silencing of the early expressed gene *TAZ1* (*ZPT3-3*) involved in *tapetum* development, produces abnormal pollen grains which are unable to germinate and to grow a normal pollen tube [50]. In the same species, silencing of the meiosis-associated gene *MEZ1* (*ZPT2-5*), yields plants producing tetracolporate pollen grains with increased DNA content that lead to premature seed abortion after pollination and fertilization [51]. Homologues for several genes that encode these ZnF transcription factors are present in the grapevine genome; and even when their function in pollen development has been not established, it is tempting to speculate a similar role to that ascribed to *ZPT* genes from petunia. Since they code only the protein moiety of the respective transcription factors, the Zn requirement to assemble the DNA binding domains could also explain the observed effect of Zn-deficiency on grapevine reproductive development [9].

### Variation in PFD rates induced by exogenous hormone application seems to be related to modification of *VvBOR3* and *VvBOR4* expression level and changes in the B concentration in floral tissues

In grapevine, fruit set seems to be regulated by hormonal changes taking place early after pollination and fertilization. Down-regulation of ABA synthesis by repressing *VvNCED1* gene expression and the onset of GA and auxin synthesis by inducing *VvGA20ox* and *VvASB1* genes, are key events occurring before fruit initiation [20]. Also, it is well documented that exogenous GA application allows fruit set without fertilization, leading to PFD in several grapevine cultivars [19,21–23]. A cross-talk between GA and auxin signaling has been suggested as the mechanism underlying the GA-induced PFD. In this model, the up-regulation of *VvDELLA* genes by GA application precedes the down-regulation of the auxin-related genes *VvIAA9* and *VvARF7* which encode transcription factors involved in negative regulation of fruit set initiation [24]. Taking this into account, in this work, inflorescences of *Vitis vinifera* cv. Carménère at pre-anthesis stage were exposed to GA and ABA applications. As expected, GA induces a significant increase in PFD rate, however ABA has the opposite effect and a reduction in PFD was observed. When compared to control plants, changes in PFD rates were not associated to modifications in abnormal pollen rates, indicating that other causes than pollen morphology are affecting its germination capability under these conditions.

Pollen tube development depends on cell wall synthesis and assembly. Dimers of the polysaccharide RG-II, cross-linked and stabilized by borate ester bonds are required for cell wall maintenance [11]. Therefore, B is essential for pollen germination and pollen tube growth [52–55] and it has been reported that B requirement in reproductive tissues is higher than in vegetative tissues [42]. B concentration in floral tissues from Carménère plants was affected in response to hormonal applications. While a marked reduction in B content was determined for GA-treated plants, exogenous ABA increased B concentration in flowers. As foliar B concentration in treated plants was maintained in the range of 55–75 ppm, the changes observed in floral B content should be caused by variation in B mobility and accumulation in this organ. Mechanisms involving different protein channels and efflux transporters are set up under B-limiting conditions in order to supply this element to aerial tissues including reproductive organs [41,42]. BOR efflux transporters seem to play a key role in B mobilization and distribution. Genes coding for such transporters have been identified and characterized in several plant species including the monocotyledonous *Oryza sativa* (*OsBOR1-OsBOR4)*, *Hordeum vulgaris* (*HvBOR2*) and *Triticum aestivum* (*TaBOR1*.*1-TaBOR1*.*3*, *TaBOR2*) as well as in the dicotyledonous *Arabidopsis thaliana* (*AtBOR1–AtBOR7*) [42]. In *Arabidopsis thaliana*, B uptake from the soil is enhanced by up-regulation at the transcriptional level of the *AtNIP5;1* gene which encodes a protein channel from the major intrinsic proteins (MIPs) family, and is localized in the plasma membrane of epidermal, cortical and endodermal cells [56]. B is relocated to aerial tissues by xylem loading through the AtBOR1 and AtBOR2 efflux transporters. Their respective genes are expressed and proteins are targeted to the plasma membrane in endodermis and root pericycle cells in a polar coordinated way [41,57–59]. The role of AtBOR1 transporter in xylem loading is essential for normal shoot and reproductive development under low B conditions. Supporting this, *Arabidopsis thaliana bor1* mutants show problems with expanding rosette leaves and seed set, whereas overexpression of the *AtBOR1* gene improves shoot development and fertility [60]. A second NIP channel, AtNIP6;1, expressed in the nodal region of the shoots, has been suggested to be involved in the xylem to phloem transport and B delivery to developing shoot tissues [61] whereas B loading to reproductive organ and tissues involves AtBOR6 transporter [41]. On the other hand, when *A*. *thaliana* plants are exposed to high B supply, *AtNIP5;1* gene expression is down-regulated [56], AtBOR1 is subjected to post-transcriptional regulation by endocytosis, polyubiquitination and proteolytic degradation [62], then following the induction of *AtBOR4* encoding an efflux transporter that removes B from epidermal cells to soil to confer tolerance to B toxicity [63]. A slightly different mechanism has been described for the monocotyledonous *Oryza sativa* where the transporter coded by *OsBOR1*, the rice closer homolog of *AtBOR1*, is involved in both, B uptake into the roots cells and xylem loading [64], and B is transported to reproductive tissues by OsBOR3 and OsBOR4 transporters [42,65].

In a previous work, a family composed of six genes encoding putative BOR-type transporters was identified in the grapevine genome; and one of them, *VvBOR1*, was fully characterized. The encoded efflux B transporter shares high identity with AtBOR1 containing specific amino acidic residues involved in polar localization and polyubiquitination. Furthermore, *VvBOR1*gene was determined as the grapevine orthologue of *AtBOR1* gene by complementation analysis in *Arabidopsis thaliana bor1* mutant [36]. Although this gene was predominantly expressed in roots, an induction of its transcriptional activity was also observed in flowers at anthesis [36]. The expression profile for the six *VvBOR* genes throughout reproductive development in Carménère cultivar was determined in this work. When compared with *VvBOR1*, higher transcriptional levels in reproductive tissues were detected only for *VvBOR3* (related to *AtBOR1*) and *VvBOR4* (related to *AtBOR4*) genes. *VvBOR3* is strongly expressed in pollen-depleted flowers at anthesis and in developing berries displaying a similar expression pattern to *OsBOR3*, which is highly expressed in ovaries and in embryos during seed development [65]. On the other hand, *VvBOR4* is specifically expressed in anthers and pollen, similar to the *OsBOR4* expression profile. Interestingly, the participation of the *OsBOR4* encoded transporter in pollen germination and pollen tube growth has been well established. Homozygous insertional mutants for this gene (*Osbor4* mutants) exhibit normal floral organ development, but produced pollen grains unable to develop pollen tubes when they reach stigmas of wild type plants [65].

*VvBOR3* and *VvBOR4* expression was modified in response to exogenous ABA and GA,. Down-regulation of both genes was observed in response to GA application either in flower at anthesis as well as in fruits at setting while up-regulation by ABA treatment was significant only for *VvBOR4* at anthesis. The above results, together with the observed modification of flower B content in hormone-treated plants, suggest that *VvBOR3* and *VvBOR4* could play similar roles to their rice counterparts *OsBOR3* and O*sBOR4* in mobilizing B to floral tissues, being directly involved in pollen germination and pollen tube growth. This assumption agrees with the reported effect of pre-bloom GA application on pollen tube growth in grapevine that affects the pollen tube penetration into ovarian tissues by an unknown mechanism [66]. Therefore, a combined mechanism involving inhibition of pollen germination by reduction in B content in floral tissues and the stimulation of fruit setting by down-regulation of genes encoding fruit initiation repressors could be operating in GA-induced PFD.
